# Buffalo Medical and Surgical Association

**Published:** 1882-06

**Authors:** C. M. Daniels

**Affiliations:** Secretary


### Buffalo Medical and Surgical Association.

*Regular Meeting, Tuesday Evening, May 2, 1882.*

President, Dr. A. R. Davidson, in the chair. Dr. C. M. Daniels, Secretary.

Present—Drs. Cronyn, Rochester, Harvey, Brecht, Briggs, Hartwig, Daggett, Runner, Moody, Storck, Bartlett, Granger, Mynter, Mann and Howe, and by invitation Drs. Fell, Coakley and Crego.

The minutes of the last meeting were read and approved.

*Essay*—By Dr. C. M. Daniels, subject: "An Objection to Listerism." The ground taken being that great reduction of temperature is consequent upon the use of the spray, as demonstrated by a series of experiments, showing a reduction of from 26° to 33°. (Extracts from the paper appear in this number of the Journal under "Original articles.")

*Discussion*—Dr. Rochester stated that the idea as applied was entirely new to him, and endorsed the grounds of objection. He spoke further regarding the rapidity with which carbolic acid is absorbed when in dilute solution, and of its sometimes causing disquamative nephritis; and also referred to the extreme uses to which it is sometimes put in uterine applications, &c., which he thought demanded great caution.

Dr. Cronyn endorsed the paper and objections raised, and thought the manner of demonstration original. He considered that "Listerism" is practically doomed, but that it has been the means of improving surgical dressings in a great degree.

Dr. Hartwig also considered the objection well taken.

Dr. Moody wished to thank the essayist for the idea advanced.

Dr. Bartlett spoke in general regarding the use of carbolic acid which he has discarded altogether and referred to the tests as tried in New Orleans several years ago, where one section of the city was set apart and disinfected with it during an epidemic, but with very unsatisfactory results. He also expressed the opinion that the only true value of "Listerism" is in the improved surgical dressings.

*Voluntary Communications*—Dr. Hartwig presented a patient having a very peculiar condition of the skin, which created much interest, and was commented upon by Drs. Rochester, Mynter, Bartlett and Cronyn, the prevailing opinion being that it was urticaria syphilitica.

Dr. Howe spoke upon the treatment of purulent discharge from the middle ear. He had often recommended that powdered alum should be blown into the ear, which should be afterward thoroughly syringed. A recent experience, however, had taught him that this treatment was not unattended with danger. Alum may produce a coagulum, notwithstanding the careful syringing of the ear as directed, which cannot easily be removed. He also related a case and showed specimen.

Dr. Fell raised the question of vaccination, stating that he had in several instances used the Bovine virus on the slips without success, but a re-vaccination with humanized virus was uniformly successful.

Dr. Bartlett referred to the timidity of physicians in using anything but Bovine virus, and thought that physicians should be justified in using humanized virus when carefully selected under personal supervision.

Dr. Rochester held to the opinion that some of the Bovine virus obtained was inert, or unprotective, especially when it produced unusual inflammatory action; and that much was obtained before the vesicle was in proper condition.

Dr. Cronyn seconded Dr. Rochester's remarks and believes good humanized virus to be recommended, especially if only one remove from the animal.

Dr. Hartwig concurred in the same opinion.

Dr. Briggs thought it imprudent to vaccinate largely without using the Bovine virus, and related his experiences during the past year as Health Physician to the effect that in general it had given satisfaction when obtained from reliable sources.

*Prevailing Diseases*—Measles and scarlatina, and Dr. Bartlett reports several cases of diphtheria.

*Miscellaneous Business*—The President gave statistics of the attendance of members of the Association for the past three years and commented upon the apparent want of interest manifested. He thought that the inconvenient location of the Association Rooms prevented the attendance of many physicians and suggested the obtainment of suitable rooms in some more central part of the city. He also spoke strongly in favor of more fully carrying out the Rules and By-Laws of the Association.

Remarks were made by Dr. Howe, who presented the following resolution and moved its adoption:

*Resolved,* That the Board of Trustees be requested to procure, if possible, a suitable room for the meetings of this Association in such location as shall be most convenient for a majority of the members.

Dr. Bartlett endorsed the resolution and spoke in favor of it.

Dr. Briggs seconded the motion.

Dr. Rochester gave some interesting reminiscenses of the early days of the Association and of the discussions upon subjects then introduced, and thought much interest might be awakened if the custom was revived. Regarding the removal to other quarters, he would endorse the action of the Society and should they at any time wish to return to their present location, the College Faculty would, doubtless, again welcome them.

Dr. Cronyn was of the opinion that the lack of attendance was from indifference, and seconded the effort for removal if it promised a better future. He also related some experiences of the past when discussions sometimes waxed warm and were the features of the meetings, and kept up the attendance and interest. He thought free discussion should be invited and encouraged.

Dr. Howe thought that promiscuous discussion without previous preparation and limited time would introduce much which was of little, if any, professional importance, and that it would evidently be better to confine speakers to certain restrictions. This was not concurred in by Dr. Cronyn, who remarked that, as it was the intention to publish the proceedings, he thought that each speaker would confine himself to subjects in question and under the ruling of the chair and would feel more free to reply under such circumstances; at least such had been the past experience of the Association.

The motion for the adoption of the resolutions was unanimously carried.

The President stated that in his opinion the July and August meetings should be suspended, which, upon motion, was carried.

Adjourned.

C. M. Daniels, *Secretary.*

